# Tailoring for Health Literacy in the Design and Development of eHealth Interventions: Systematic Review

**DOI:** 10.2196/76172

**Published:** 2025-09-02

**Authors:** Jan Wessel Hovingh, Cynthia Elderson-van Duin, Derek A Kuipers, Yanda van Rood, Geke D S Ludden, Denise J C Hanssen, Judith G M Rosmalen

**Affiliations:** 1 Department of Psychiatry, University Medical Center Groningen, University of Groningen, Hanzeplein 1, Groningen, 9713 GZ, The Netherlands, 31 (0)50 361 61 61; 2 Department of Interaction Design, Department of Design, Production and Management, University of Twente, Enschede, The Netherlands; 3 Future Design Center, Faculty of Tech and Design, NHL Stenden University of Applied Sciences, Groningen, The Netherlands; 4 Department of Psychiatry, Leiden University Medical Center, Leiden University, Leiden, The Netherlands; 5 Department of Internal Medicine, University Medical Center Groningen, University of Groningen, Groningen, The Netherlands

**Keywords:** eHealth, tailoring, health literacy, design, design rationale, development, PRISMA

## Abstract

**Background:**

Tailoring is an important strategy to improve uptake and efficacy of medical information and guidance provided through eHealth interventions. Given the rapid expansion of eHealth, understanding the design rationale of such tailored interventions is vital for further development of and research into eHealth interventions aimed at improving health and healthy behavior.

**Objective:**

This systematic review examines the use of health literacy concepts through tailoring strategies in digital health interventions (eHealth) aimed at improving health and how these elements inform the overall design rationale.

**Methods:**

A systematic search of PubMed, PsycINFO, Web of Science, and ACM databases yielded 31 eligible randomized trials that focused on adult health improvement through eHealth interventions. Eligible studies compared tailored versus nontailored eHealth interventions for adults, excluding non-English papers and those addressing solely readability or targeting populations with accessibility barriers. Data extraction focused on study characteristics, health literacy components, tailoring methods, and design rationales, with study quality evaluated using Quality Assessment for Diverse Studies (QuADS) by independent reviewers.

**Results:**

Most interventions applied both cognitive and social health literacy concepts and predominantly used content matching as a tailoring strategy. Of all studies using content matching, most used one or more supporting theories as well as end-user data to inform the content matching. While choices for individual intervention components were mostly explicated, detailed descriptions of the design process were scarce, with only a few studies articulating an underlying narrative that integrated the most important chosen components.

**Conclusions:**

While tailored eHealth interventions demonstrate promise in enhancing health literacy and the trial design of the interventions overall was of good quality, inconsistent documentation of design rationales impedes replicability and broader application of the used eHealth concepts. This calls for more detailed reporting on the design choices of the intervention in efficacy studies, so that reported outcomes can be easier connected to choices made in the design of the eHealth intervention.

## Introduction

In recent years, the design, development, and implementation of digital health interventions, or eHealth interventions, has transformed the landscape of health care and health care delivery [1]. Through these interventions, a wide range of technologies, including mobile apps, portal websites, serious games, and wearables, have been conceived, tested, and implemented in various health care settings. These technologies have shown great promise to enhance accessibility [2] and usability of various health care concepts and services [3]. However, despite the potential benefits of eHealth interventions in improving health outcomes, their impact is not equally distributed. Many end users encounter difficulties in comprehending and using the information provided by these interventions, leading to underperformance compared with their anticipated potential [4].

One of the most important mechanisms associated with health outcomes is health literacy [5]. Health literacy has been defined as people’s knowledge, motivation, and competencies to access, understand, appraise, and apply health information in order to make judgments and take decisions in everyday life concerning health care, disease prevention, and health promotion [6]. Initially, health literacy was defined as a mainly cognitive skill focused on information processing, including understanding, reading, and knowledge of health information. Recent perspectives have adopted a more comprehensive approach to the concept by including the significance of social skills and executive capacities, such as goal-setting, taking action, confidence, motivation, beliefs, and role expectations [7,8]. Most likely, health literacy in its broadest sense impacts the use and effectiveness of eHealth interventions.

One strategy to increase the impact of eHealth interventions is to tailor these interventions toward the specific needs and capacity of the end-user, ensuring that desired knowledge and behavior cues are transferred in the most suitable manner. Tailoring refers to the optimization of communication and functionality based on assessable characteristics unique to that individual. Choosing tailoring tactics, guided by theoretical and empirical evidence, can lead to better understanding and uptake of information and subsequently increased efficacy of the intervention [9].

Regarding health literacy, especially the strategy of content matching, seems important. Named “the crux” of tailoring [10], content matching attempts to direct messages to individuals’ status on key theoretical determinants (such as knowledge, outcome expectations, normative beliefs, efficacy, or skills) of the behavior of interest [9]. Ideally, these assessable determinants are established by an evaluative framework, supported by the use of specific supporting theories such as the Transtheoretical Model or the Social Cognitive Theory [10].

Indeed, tailoring for health literacy could follow 2 main conceptual approaches. The first one involves optimizing communication and functionality, focusing on the cognitive capacity of an end-user: in short, an end-user needs to understand and become susceptible to the provided health-related information to act accordingly [9]. The second involves focusing on the social capacity of an end-user. The end-user is advised or encouraged to show certain health-related behaviors [11]. Of course, these 2 approaches are not mutually exclusive and may both be present in an eHealth intervention at the same time, ideally both supporting the same targeted behavior.

The question is how these tailoring strategies, especially content matching, can help to translate the cognitive and social health literacy concepts into a usable and assessable eHealth intervention that meets the desired outcomes. This can be done by establishing the design rationale of the intervention: a representation of the reasoning behind the design of that intervention [12]. It explains choices such as type of technology, content, and usability from an underlying story of the concept that ties together the different elements of the solution into one coherent argument for the solution as a whole [13].

Unfortunately, there is a lack of an overview of how health literacy concepts and how tailoring strategies are used in eHealth interventions, including how health literacy and tailoring are combined. Several reviews have shown that tailored interventions are important for increasing health literacy itself [4,14,15]. However, they did not clarify how social and cognitive health literacy specifically contributed to the tailoring process. Moreover, they did not describe in what way the forthcoming tailoring strategies added to the design rationale of the intervention [4].

Therefore, in this study, we aim:

1. To provide a structured overview of the use of cognitive and social health literacy concepts in tailored eHealth interventions that aim to improve health.
2. To provide a structured overview of tailoring strategies used in eHealth interventions that aim to improve health.
3. To explore how these cognitive and social health literacy concepts and tailoring strategies contributed to the design rationale of eHealth interventions.

## Methods

### Overview

This systematic review was executed and reported according to PRISMA (Preferred Reporting Items for Systematic Reviews and Meta-Analyses) criteria for systematic reviews [16]. The review was registered with PROSPERO (International Prospective Register of Systematic Reviews; 225731). The review has been slightly altered after registration in PROSPERO in terms of focus and scope. While the original goal also included studying eHealth intervention efficacy and health literacy-related outcomes, the selected studies varied too much in terms of setup, execution, dosage, and population to draw useful overarching conclusions. Instead, we decided to focus on the design rationales of the eHealth interventions.

### Search Strategy

Studies were identified by searching PubMed (MEDLINE), PsycINFO, Web of Science, and Association for Computing Machinery (ACM) for publications before June 2021. The search strategy was based on previous health literacy reviews [4,17]. For all databases, search strings were compiled using 3 main concepts: eHealth, Health Literacy, and Tailoring. For the string “eHealth”, variations of the term eHealth as well as alternative descriptions for digital health interventions (ie, mobile health [mHealth], internet intervention, and online intervention) were used. For the search string “Health Literacy”, both cognitive (knowledge, competence, and health literacy) and social (patient activation and motivation) health-related terms were used, based on the search strategy from the European Health Literacy Survey (HLS-EU) consortium [4]. For the string “Tailoring”, the search strategy of an earlier review was adapted [17], which included terms such as “tailoring”, “personalization*.”* The 3 resulting search strings were combined using the Boolean operator “AND”. The queries were then adjusted and optimized for any of the databases. The complete strategy per database can be found in Table S1 in Multimedia Appendix 1.

### Eligibility Criteria

When selecting relevant literature, we included (1) trials with at least one control group; (2) eHealth studies aimed at improving health, such as studies on patient education, decision support, risk assessment, health behavior change, treatment, or self-management of physical and psychological illnesses; (3) studies focused on adults; (4) studies that measured health literacy (cognitive, social); (5) studies that compared a tailored eHealth intervention with a nontailored intervention. We excluded all studies that (1) were not written in English; (2) targeted populations lacking basic accessibility requirements (functionally illiterate, deaf, or blind); (3) focused on cultural tailoring; (4) were only focusing on wording and readability of written content; (5) had active human involvement in the intervention, other than the patient himself; (6) only compared differences in medium type (eg. printed media compared with online media).

### Study Selection

After extraction from the databases, all identified studies were uploaded to Covidence Systematic Review software (Veritas Health Innovation Ltd) for screening and removal of duplicates. First, titles and abstracts of uploaded studies were independently screened by 2 reviewers for the eligibility criteria (JH and CE). Any disagreements on the title and abstract screening were negotiated by accessing the full text to determine its appropriateness. In the second stage, full-text papers were further screened for the eligibility criteria. Any disagreements in all previous steps were extensively discussed and resolved by the reviewers (JH, CE) or, when they were unable to agree, by a third reviewer (JR, DK).

### Data Extraction

All data were extracted independently by 2 reviewers (JH, CE for tailoring questions; JH, DK for design rationale questions, Quality Assessment for Diverse Studies [QuADS] by JH and CE).

#### General Characteristics

We extracted the first author, year of publication, country of origin, titles, and targeted illness or targeted behavior.

#### Health Literacy Concepts

When extracting information on health literacy, we focused on descriptions of both cognitive as well as social approaches to health literacy concepts in the intervention and extracted the reported details on these concepts.

#### Tailoring Strategies

Tailoring strategy types were categorized into Personalization, Feedback, and Content Matching, plus their corresponding subsets [10]. We also extracted information on the theoretical framework for content matching in the form of reported supporting theories and whether personal data of end-users was used for content matching.

#### Design Rationale

To establish the design rationale of the intervention, we looked at the intervention itself, its intended goals, its components, the design process, and the reasoning behind the choices made in that process.

We therefore categorized (1) the medium (website, app, email, or message service), (2) the hardware (desktop, mobile app, wearable, or tablet), (3) the components (eLearning, Game or Game mechanics, Diary logging, or Questionnaires). We further extracted (4) hypotheses concerning envisioned or desired workings of the intervention connected to its intended goals; (5) information on the design process (if any such process was reported and whether a design method was used); and (6) the reasoning related to the choices for the health literacy, tailoring, and design characteristics of the intervention. We used a classification of 3 types of reasoning: pragmatic (based on ease of accessibility or availability), theoretical (based on relevant scientific evidence in other contexts) and contextual (based on evidence from the end user’s context) and (7) whether an underlying story was provided which ties together the different elements of the solution.

### Quality and Bias Assessment

We only included randomized controlled trials, and while this strategy was initially intended to study efficacy, we did choose to include the study quality review. Quality and bias assessment was based on the criteria of QuADS [18] by 2 independent reviewers using Covidence. Any disagreements were resolved by discussion between the reviewers (JH and CE) or, when they were unable to agree, by a third reviewer (JR).

## Results

### Search Results

**Figure 1.**
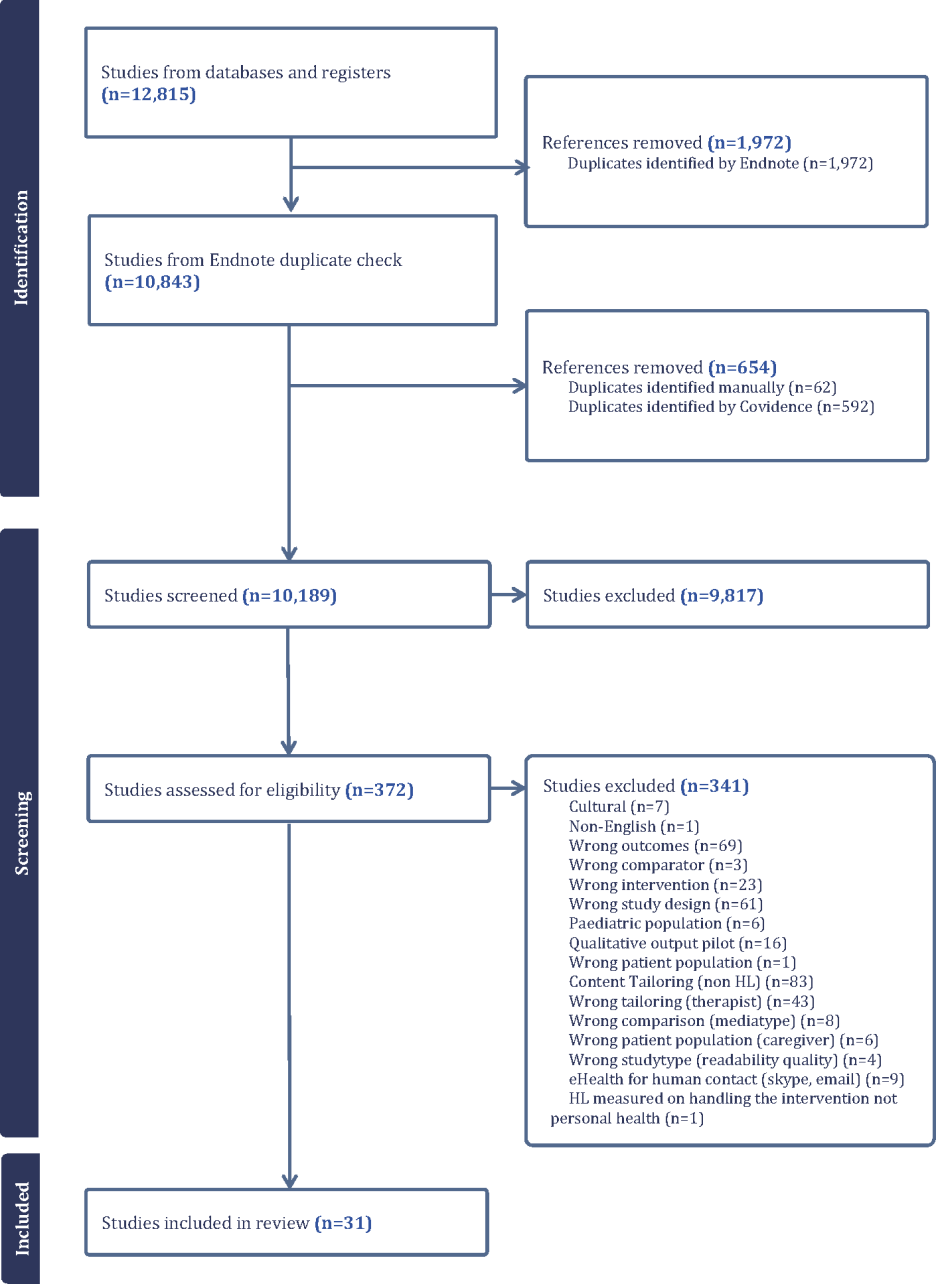
Preferred Reporting Items for Systematic Reviews and Meta-Analyses flow chart.

The search yielded 12,815 results (Pubmed: 3308; Psychinfo 1916; Web of Science 3188, ACM 4403). EndNote was used to discard duplicates for all databases, after which 10,843 references were imported into Covidence. Using Covidence, a further 592 duplicates were removed before screening. Eventually, the screening resulted in 31 studies that were eligible for data extraction and analysis (Figure 1).

### General Characteristics

Of the 31 selected papers [19-49], 16 studies were from the United States [20,22,23,26-30,33,36,37,41-45], 4 from the Netherlands [25,31,39,40], 3 from Canada [21,24,32], 3 from Australia [19,47,49], and 1 each from New Zealand [38], Germany [34], Switzerland [34], and Hong Kong [48]; 1 study used an international sample [35].

Only 2 papers were published before 2010 [23,37]. Between 2010 and 2015, 11 papers were published [19,26,28,30,36,38,39,41,45-47], with 4 papers in 2015 alone [36,38,46,47]. Between 2016 and 2020, 17 papers were published [20-22,24,25,27,29,31-35,40,42-44,49], with 9 in 2020 alone [22,24,27,29,31,32,40,42,43]. For 2021, 1 paper was included [48], but this may be partly due to the extraction date.

Most of the interventions addressed a single clinical or behavioral topic, 1 addressed 2 topics [46], and 1 addressed 4 topics simultaneously [44].

Of the screened interventions, 52% (16) were targeted at specific illnesses, complaints, or symptoms, such as cancer (5) [19,27,33,45,47], diabetes (4) [22,34,43,46], cardiovascular diseases (4) [29,31,37,38], back pain (3) [28,36,46], menopausal symptoms (1) [23], and migraine (1) [26].

The remaining interventions (15) were focused on behavioral, preventative, or informative health-related activities such as food intake (3) [20,24,44], smoking (3) [25,44,49], physical activity (2) [40,44], STIs and prevention (2) [39,48], weight management (1) [44], or other preventative care (4) [30,35,41,48]. Two interventions informed end users on specific medical procedures, such as medicine adherence (1) [21], breast reconstruction (1) [42], and one intervention focused on coping strategies for domestic violence [32] (Table S1 in Multimedia Appendix 2).

### Health Literacy Concepts

A total of 18 studies [19,23-33,36,39,42,43,45,48] described interventions that used both cognitive and social aspects of Health Literacy concepts. 6 studies [20,35,41,44,46,47] only contain interventions that take a cognitive approach to Health Literacy, while 7 studies [21,22,34,37,38,40,49] only aim to convey one or more social components of health literacy. Some of the health literacy concepts are used to explicitly convey knowledge or awareness on a certain health-related topic with very little or no behavioral cues [19,20], while others use a more practical approach and focus on the social component of health literacy by helping end users develop new behavioral patterns [21,22]. Interestingly, while all included interventions make use of health literacy related concepts, only a very few studies explicitly mention health literacy or a derivative such as food literacy [50], as a concept used to inform the design of the intervention (Table 1).

**Table 1.** Tailoring strategies and health literacy concepts.

Author	Tailoring strategy types	Supporting theory or theories	Personal data used for content matching	HL^a^ cognitive	HL^a^ social
Barnabei et al [23]	Content Matching, Feedback (Descriptive)	None reported	Yes	Explanation of Hormone therapy	Customized questions to ask the provider about HT^c^
Bomfim et al [24]	Content matching, personalization (identification, raising expectation, contextualization), feedback (descriptive, comparative, evaluative)	Self-Affirmation Theory, Self-Determination Theory, and Food Literacy Theory	Yes	Develop the knowledge and awareness of nutrition and food	“Make informed food decisions when grocery shopping.”
Bommele et al [25]	Content matching, personalization (identification) feedback (descriptive, evaluative, comparative)	Self-Affirmation Theory and Self-Efficacy Theory	Yes	Emphasized the cons of smoking and the pros of quitting	Increase receptivity to antismoking information
Boyd et al [29]	Content matching, personalization (contextualization)	None reported	Yes	Patient narratives about the importance of DAPT	Helping the patient plan and overcome the common barriers to medication adherence
Bromberg et al [26]	Content matching, feedback (evaluative, comparative), personalization (identification, contextualization)	Cognitive Behavior Therapy	Yes	Migraine-specific knowledge, medication safety	Migraine self-management skills, emotional coping, communication skills
Burgermaster et al [20]	Content matching, feedback (descriptive, evaluative, comparative)	Health Literacy, Nutrition Literacy, and Social Cognitive Theory	No	Vicarious learning about caloric contents of food	None reported
Carter-Harris et al [27]	Content matching, personalization (contextualization)	Conceptual Model on Lung Cancer Screening Participation: Health Belief Model Precaution Adoption Process Model	Yes	Lung health knowledge, knowledge of risk factors for lung cancer, knowledge of option of lung cancer screening; knowledge of risks and benefits of lung cancer screening	Tailored summary that individuals can use to guide a discussion with their clinician
Chiauzzi et al [28]	Content matching, personalization (contextualization), feedback	Cognitive Behavior Therapy	Yes	Motivational enhancement	Collaborative decision making with health professionals 2) CBT to improve self-efficacy, manage thoughts and mood, set clinical goals, work on problem-solving life situations, and prevent pain relapses; 3. wellness activities to enhance good sleep, nutrition, stress management, and exercise practices.
Côté et al [21]	Content matching, feedback (comparative), personalization (identification)	Social Learning Theory, Behavior Change techniques	No	None reported	Reinforcing self-management skills required for medication intake. The sessions aimed to help users incorporate the therapeutic regimen in their daily routine, cope with medication side effects, handle situations or circumstances that could interfere with medication intake, interact with health care professionals, and mobilize social support. The learning objectives included strengthening various capacities such as self-motivation and self-monitoring
Dingle and Carter [49]	Content matching, feedback (comparative)	Elaborated Intrusion Theory of Desire for Smoking	No	None reported	Psychoeducation about smoking and its triggers, particularly emotional states and cravings Learning how to use music to mediate negative affective triggers that may lead to smoking behavior
Drieling et al [30]	Content matching, personalization (contextualization)	SMCR communication model, Social Cognitive Theory, Transtheoretical Model	Yes	Education on risk factors, knowledge, and attitudes concerning osteoporosis	Tutorials for healthy behavior concerning osteoporosis
Engelen et al [31]	Content matching, personalization, feedback	I-change Model	Yes	Knowledge, awareness, Intention, Attitude, Self-Efficacy CVD, Coping CVD, Boundaries in daily life CVD, Lifestyle CVD, Healthy nutrition CVD, Physical Activity CVD, Interaction with Health Professionals	Habits, skills, Norm, Intention, Attitude, Self-Efficacy CVD: Coping CVD: Boundaries in daily life CVD: Lifestyle CVD: Healthy nutrition CVD: PA CVD: Interaction with Health Professionals
Flight et al [19]	Content matching, personalization (identification, contextualization)	Preventive Health Model, Precaution Adoption Process Model	Yes	Educational content with “generalized risk information (ie,>50 years, certain bowel conditions, and family history”	Messages tailored to an individual user’s decision stage for screening and responses to PHM, self-efficacy, and fecal aversion variables
Ford-Gilboe et al [32]	Content matching, personalization (identification, raising expectation, contextualization), feedback (comparative, evaluative)	Danger Assessment Calendar	Yes	Increase their awareness of safety risks	Reflecting on (.) plans for their relationships and priorities, a personalized detailed action plan of strategies and resources for addressing their safety and health concerns
Fowler et al [33]	Content matching, personalization (contextualization), feedback (descriptive, comparative, evaluative)	None reported	Yes	Informing participants of the extent to which behavior change could reduce their risk.	“It targets self-efficacy by suggesting specific strategies for changing behavior.”
Gimbel et al [22]	Content matching, personalization (contextualization), feedback (descriptive, evaluative)	Patient Activation Measure Summary of Diabetes Self-Care Activities	Yes	None reported	Behavioral messaging concerning nutrition, home monitoring, physical activity, blood pressure, foot care, medications, smoking, glucose control, and general behavioral reinforcement.
Höchsmann et al [34]	Content matching, feedback (descriptive, evaluative)	Self-Determination Theory	Yes	None reported	“In the game, regular PA is rewarded with water or building materials that are needed to restore the garden and proceed in the storyline. In-game workouts consist of 130 variations of strength, endurance, balance, and flexibility exercises whose execution, as well as daily PA, is tracked via the phone’s sensors (camera, accelerometer, and gyroscope).”
Hopkin et al [35]	Content matching, feedback (evaluative)	Subjective Expected Utility Theory	Yes	Knowledge of benefit and harm outcome of statins according to their own preferences.	None reported
Irvine et al [36]	Content matching, personalization (identification, contextualization), feedback (descriptive, evaluative)	Social Cognitive Theory, Theory of Planned Behavior	Yes	General aspects of pain and pain management	Pain self-care activities in 4 categories (rest and relief, mindfulness, general fitness, and back pain-specific stretching and strength exercises), cognitive and behavioral strategies to manage and prevent pain (eg, controlling fear of pain, mindfulness and relaxation, use of heat and ice, over-the-counter medications, benefits of staying active), and instructional videos on specific strength and stretching exercises tailored by job type (sitter, stander, driver, lifter)
Kukafka et al [37]	Content matching, feedback	Self-Efficacy Change Theory	Yes	None reported	“Seeking help in responding to AMI symptoms specific to each dimension of self-efficacy scale”
Maddison et al [38]	Feedback (descriptive), personalization (contextualization)	None reported	No	None reported	Regular exercise prescription, provision of behavior change strategies
Mevissen et al [39]	Content matching, personalization (identification, contextualization), feedback (evaluative)	AIDS Risk Reduction Model, Extended Parallel Process Model, Motivational Interviewing	Yes	Addressing perceived risk of STI^b^ infections within established relationships; Targeting normative beliefs associated with condom use and STI testing in established relationships	self-efficacy and skills related to condom use and STI testing
Middelweerd et al [40]	Content matching, personalization (contextualization), feedback (descriptive, comparative, evaluative)	Health Behavior Theory	Yes	None reported	Coaching on sports participation, taking the stairs, or active transport.
Milan and White [41]	Content matching	Transtheoretical Model	Yes	“Five educational modules, each corresponding to the five stages of readiness for meeting folic acid multivitamin recommendations.” - “Each module consisted of 4 Web pages, designed to be read over the course of 4 weeks in a 5- to 10-minute block of time”	None reported
Politi et al [42]	Feedback (Evaluative)	None reported	No	breast reconstruction knowledge	Asking the right questions during a consultation
Sittig et al [43]	Content matching, personalization (contextualization), feedback (evaluative)	Social Cognitive Theory, Fogg Behavior Model, Persuasive Technology	No	Knowledge on type 2 diabetes	Self-efficacy, Self-care for type 2 diabetes, goal setting
Valle et al [44]	Content matching, personalization (identification, raising expectation), feedback (comparative)	Social Cognitive Theory, Theory of Planned Behavior	Yes	Feedback messages on health risk assessment	None reported
Vernon et al [45]	Content matching, personalization (identification), feedback (evaluative)	Transtheoretical Model	Yes	Knowledge about CRCS, CRC risk perception, Awareness of CRC as a serious problem	Self-efficacy, Goal Setting, Getting CRCS
Weymann et al [46]	Content matching, personalization	Summary of Diabetes Self-Care Activities Measure, Avoidance Endurance Model	Yes	Presenting and testing information on T2D and CLBP, self-management education	None reported
Wilson et al [47]	Content matching	Preventive Health Model	Yes	Information sheet; tailored messages; consumer information booklet	None reported
Wong et al [48]	Content matching, feedback (evaluative)	Health Literacy, Health Belief Model, Harvard Cancer Risk Index, Continuum of Conflict and Control Theory	Yes	Disseminate knowledge about STIs, cervical cancer, and condom use.	Take action regarding condom use, communication and negotiation about condom use, and sexual coercion in daily life

a HL: health literacy

b STI: Sexually Transmitted Infection

c Hormone Therapy

### Tailoring Strategies

Content Matching (29 studies, [19-37,39-41,43-49]) was found in almost all studies, followed by Feedback (24 studies, [20-26,28,31-40,42-45,48,49]) and Personalization (21 studies, [19,21,22,24-33,36,38-40,43-46]); this includes 15 studies using all 3 tailoring strategies [21,22,24-26,28,31-33,36,39,40,43-45]. In one study, we also could discern all subsets [24]. 13 studies used 2 strategies [19,20,23,27,29,30,34,35,37,38,46,48,49] and 3 used only one [41,42,47]. Content Matching was often guided by personal data (25 studies, [23-37,39-41,44-48]) and by theoretical frameworks (28 studies [19-22,24-28,30-32,34-37,39-41,43-49]); only 2 studies [20,48] explicitly mention Health Literacy as a guiding principle for tailoring, while 2 name Food Literacy as a supporting theory [20,24]. Of all interventions, 13 used one supporting theory, 7 used 2 theories, 5 used 3 theories, and one intervention used 4 supporting theories. 5 interventions did not report any use of supporting theories. Of the interventions that did not use any supporting theories, 3 relied on personal data from end users for content matching. Apart from 7 theories that were reported twice, the most prevalent supporting theories were Social Cognitive Theory (5) and the Transtheoretical Model (3) (Table 2).

**Table 2.** Design rationale and process.

Author	Rationale for HL^a^ concepts	Rationale for tailoring strategy	Rationale for technology or modules	Solid overarching reasoning for intervention	Design process	Development or design method
Barnabei et al [23]	None reported	Theoretical	Theoretical, Contextual	None reported	None reported	None reported
Bomfim et al [24]	Theoretical, Contextual	Theoretical, Contextual	Theoretical, Contextual	Reported^b^	External	None reported
Bommele et al [25]	Theoretical	Contextual	Theoretical	None reported	None reported	Intervention Mapping
Boyd et al [29]	Contextual, Theoretical	Contextual	Contextual, Theoretical	None reported
Bromberg et al [26]	Theoretical	Theoretical	Theoretical	None reported	None reported	The self-management education model by Lorig and colleagues at Stanford University
Burgermaster et al [20]	Theoretical	Theoretical	Theoretical	Reported^d^	None reported	None reported
Carter-Harris et al [27]	Contextual	Contextual	Pragmatic	None reported	External	USPSTF^c^ Lung Cancer Screening Guidelines, International Patient Decision Aid Standards
Chiauzzi et al [28]	Theoretical	Contextual	Theoretical, Contextual	None reported	External	None reported
Côté et al [21]	Theoretical	None	Theoretical, Pragmatic	Reported^e^	External	None reported
Dingle and Carter [49]	Theoretical	Theoretical	Theoretical, Pragmatic	None reported	None reported	None reported
Drieling et al [30]	Theoretical, Contextual	Theoretical	Theoretical, Pragmatic	None reported	None reported	None reported
Engelen et al [31]	Theoretical, Contextual	Contextual	Theoretical, Contextual	None reported	External	Intervention Mapping
Flight et al [19]	Theoretical	Theoretical	Theoretical	None reported	None reported	None reported
Ford-Gilboe et al [32]	Theoretical	Theoretical	Pragmatical, Theoretical	None reported	None reported	None reported
Fowler et al [33]	Theoretical	Theoretical	Theoretical, Pragmatic	None reported	External	None reported
Gimbel et al [22]	Theoretical, Contextual	Theoretical	Theoretical, Contextual	None reported	Reported	“User-Centered Design”
Höchsmann et al [34]	Contextual, Theoretical	Contextual, Theoretical	Contextual, Theoretical	Reported^f^	External	None reported
Hopkin et al [35]	Theoretical	Theoretical	Theoretical	None reported	None reported	None reported
Irvine et al [36]	Theoretical	Theoretical, Contextual	Pragmatic	None reported	None reported	None reported
Kukafka et al [37]	Theoretical	Theoretical	Pragmatic	None reported	External	None reported
Maddison et al [38]	Theoretical	Pragmatic	Pragmatic, Theoretical	None reported	None reported	mHealth Development and Evaluation Framework
Mevissen et al [39]	Contextual, Theoretical	Theoretical, Contextual	Theoretical	None reported	None reported	Intervention Mapping
Middelweerd et al [40]	Theoretical	Theoretical	Pragmatic, Theoretical	None reported	External	“A systematic and stepwise approach”
Milan and White [41]	Theoretical	Theoretical	None reported	None reported	None reported	None reported
Politi et al [42]	Theoretical, Contextual	Theoretical	None reported	None reported	Reported	International Patient Decision Aids Standards
Sittig et al [43]	Theoretical, Contextual	Theoretical, Contextual	Pragmatic, Theoretical	None reported	Reported	Interactive Health Communication Applications framework, semi structured focus group question model
Valle et al [44]	Theoretical	Theoretical, Contextual	Theoretical	None reported	None reported	None reported
Vernon et al [45]	Theoretical	Theoretical	Pragmatic	None reported	None reported	Intervention Mapping
Weymann et al [46]	Theoretical	Theoretical	Theoretical	None reported	Reported	None reported
Wilson et al [47]	Theoretical	Theoretical	Theoretical	None reported	None reported	None reported
Wong et al [48]	Theoretical, Contextual	Theoretical	Contextual, Theoretical, Pragmatic	None reported	None reported	None reported

a HL: health literacy

b “Our gameful app, Pirate Bri’s Grocery Adventure (PBGA), incorporates gameful design elements, such as challenges, personalization, and meaningful choices to motivate young adults to develop food literacy, increase awareness, and improve choices at the grocery store.”

c United States Preventive Services Task Force

d “Taken together, these theoretical foundations point to the potential for casual observational learning to occur when people are intrinsically motivated to participate in learning activities to improve nutrition literacy on a social computing platform.”

e “This program taught participants to identify which emotions were triggers for their smoking, and how music can be used as a substitute for the emotion regulating effect of smoking”

f “The game uses the self-determination theory as the theoretical framework. The self-determination theory is a widely researched theory of motivation that addresses both intrinsic and extrinsic motives for acting and has shown its utility in explaining processes that underpin exercise behavior as well as motivation to play video games. The goal of the game is to restore a decayed garden by planting trees and flowers. In doing so, the player attracts animals that used to live in the garden to come back and help with the restoration process. At the same time, the Schweinehund, the game’s adversary, is kept in check. In German, “innerer Schweinehund” (inner swine hound) refers to the weak or lazy part of one’s nature, often regarding PA, that has to be overcome to get one’s self going. The garden setting was deliberately chosen, as its restoration stands metaphorically for the restoration of the player’s body through regular PA. In addition, it has been shown that gardening is among the target group’s preferred forms of PA and that gardening-themed games are quite popular and comprehensible to a wide range of players because of their straightforward mechanics.”

### Design Rationale

Most interventions used a website (24, [19-21,23,24,27,28,30-33,35-39,41,42,44-49]), while 7 interventions were described as an app or native application [22,24,25,29,34,40,43], 28 studies describe interventions using eLearning [19-21,23-34,36-39,41-49], 25 used a form of automated feedback [19-25,27,28,30-35,37-40,42-46,48] while 24 incorporated one or more questionnaires [19,20,23,25-37,39,41-46,48]. 11 interventions used diaries or logging [22,24,26,29,31,34,36,38,40,43,49], 5 interventions made use of a virtual agent [21,25,36,39,44], and 4 interventions used serious games or game mechanics [24,34,40,43] (Table S1 in Multimedia Appendix 3).

A clear trend in all studies is the use of eLearning. 28 studies report the use of this component to convey health-related information [19-21,23-34,36-39,41-49]. Out of the 3 studies that did not use eLearning [22,35,40], 2 did not use cognitive health literacy concepts either [22,40]. These studies relied heavily on providing automated feedback on end-user reporting and behavioral data, thus aiming to reinforce desired health-related behavior [22,40].

All studies (31) explicitly described envisioned or intended workings of the intervention by connecting the functional description of the intervention to its intended goals. Only 4 studies provided specifics concerning the design process of the intervention in the publication [22,42,43,46] while 10 studies referred to other publications for details on the design process [21,24,27-29,31,33,34,37,40]. The majority, however, did not report any details on the design process of the intervention (17). 12 studies mentioned a design method [22,25-27,29,31,38,40,42,43,45], of which 4 [25,31,39,45] referred to Intervention Mapping [51] and 2 pointed towards the International Patient Decision Aids Standards [52] as a framework for designing the intervention. Other examples of methods named were the mHealth Development and Evaluation Framework [53] and “User-Centered Design.”

In 4 studies, we could pinpoint an “underlying story” that explicitly tied the elements of the solution together [20,21,24,34]. These “stories,” however, varied significantly in detail and clarity. For example, Höchsmann et al [34] extensively explain how and why the choice of medium and components contribute to the transfer of health literacy concepts to the target group by using the metaphor used in the intervention as the underlying story—in this case the maintenance of a garden as a metaphor for living healthy—that ties the elements of the intervention together. Burgermaster et al [19-37,39-41,43-49], on the other hand, is much more succinct: the authors tie the intervention’s goal, the medium, and the health literacy content together in one sentence from a theoretical starting point.

When it comes to reasoning, a clear trend is noticeable. The vast majority of reasoning types that were used was theoretical: reasoning based on earlier research in other contexts than the research or intervention’s end user context. All but one intervention used theoretical reasoning for one or more components of the interventions. 16 of the 31 interventions use contextual reasoning to support choices for one or more of the components of the intervention [22-25,27-31,34,36,39,42-44,48] (Table 2).

### Quality and Bias Assessment

The results of the QuADS assessments can be found in Table S1 in Multimedia Appendix 4. Of the included studies in this systematic review, 26 studies (79%) were of high quality (scoring above 66%), and 7 (21%) were of moderate quality (scoring between 33% and 66%). No low-quality studies were found. The lowest score was 51%. Main areas for quality improvements were: (1) strengths and limitations critically discussed; and (2) appropriate sampling to address the research aim.

## Discussion

### Principal Findings

The goal of this review was threefold: to provide an overview of the health literacy concepts and tailoring strategies used in eHealth interventions that aim to improve health and to determine how these elements contributed to the design choices of the interventions. In all selected studies, health literacy concepts are used to inform the design of the interventions. The types of concepts and their application vary significantly, as does the choice of tailoring strategies in these interventions. Content matching is present in almost all studies, but a clear trend in applied design strategies or patterns for tailoring strategies to inform the design of the interventions cannot be distinguished.

Concerning the overview of the health literacy concepts, in all extracted papers, we can identify the use of health literacy concepts as guiding principles to aid the development of the inner workings and content of the interventions. Most eHealth interventions used both cognitive and social health literacy concepts. However, there is a broad variety in how these concepts are described, used, and translated into design choices for the intervention and how these concepts are tailored towards the end users. Considering the differences in contexts and targeted illnesses and behaviors, this is not entirely surprising, but there are no clear patterns to be found in approaching health literacy on the whole.

As for the tailoring strategies, content matching can be found in all but 2 studies [19-37,39-41,43-48] while personalization and feedback are used significantly less. Personalization and feedback have been described as mechanisms aimed at “message processing” and “self-referencing,” hinting at “creating preconditions for message processing” [9], while content tailoring is described as “the crux” of tailoring. This may explain why personalization and feedback are less common and why only 15 studies show the use of all tailoring strategies [21,22,24-26,28,31-33,36,39,40,43-45].

Only 2 studies [19,44] explicitly refer to the reporting recommendations for tailored interventions [10], in which these strategies are proposed. The way in which content matching is applied varies.

A minority of the studies reported a design process and even fewer reported a methodology for the design process of the intervention. Only 4 studies described a design process in the paper itself [22,42,43,46], although in most studies, rationales for choices for the main components of the interventions were provided. Interestingly, 2 of these interventions involved serious games [24,34]. This may be partly explained by the fact that serious games in the context of health literacy are a relatively novel approach to eHealth interventions, provoking more extensive reporting on how the product was conceived. Also, it is quite common that serious games are designed around a metaphor, which increases the chances of an overarching story that ties together the elements of the solution [54].

The main type of rationale provided for the choice of health literacy concepts, tailoring strategy, and technology was theoretical. Although a qualitative assessment on the rationales is difficult to make, choices for technology and modules would require some contextual reasoning: reasoning based on needs, affordances, and preferences of an end user’s context [55]. In half of the studied interventions in this review, such reasoning was not reported.

As all extracted studies aimed to determine the intervention efficacy, the outcomes and conclusions of these studies are focused on answering the question whether the intervention works as intended and expected. However, for insights into why these interventions work and how the combined workings of their components contribute to efficacy, it is important to know what specific reasoning lies behind the choices for concepts, tailoring strategy, and choice of technology. Reporting on these aspects is thus important for the development and improvement of new and existing interventions.

However, we did not find any indication of formal methods such as Toulmin [56] or IBIS (Issue-Based Information Systems) [57], or use of the guidelines for reporting on tailored interventions [10]. While some of the studies were conducted before the latter guidelines were made, more recent papers also did not completely adhere to the provided recommendations. Moreover, some of these recommendations can be interpreted as a call for a summarized design rationale of the intervention, rather than just a description of the study design. On the other hand, the recommendations do not move beyond describing how supporting theories translate into tailored messages. From an eHealth perspective, a reasoned choice of technology and technological components would certainly add to these recommendations.

eHealth interventions bring together both evidence-based health practices and human-computer interaction, and thus 2 highly different paradigms for design, development, and evaluation. For example, in 4 studies, intervention mapping was named as a design process. While this method does offer a well-documented evidence-based approach, it was not specifically developed for creating digital interventions and may not offer the most suitable procedural approach when designing digital interventions.

Literature calls for a deeper understanding of and appreciation for the differences in development methods to make more effective eHealth interventions and provide more useful documentation on its research [58]. Health intervention development focuses on choosing proven “mechanisms of action” or “active ingredients” [59]. Human-computer interaction, on the other hand, has a deeply rooted tradition in ethnological research and iterative design cycles where anticipated end-users are actively participating in the complete design process [55]. We have found little evidence of these types of design practices in the reporting.

### Strengths and Limitations of This Review

This review is the first review on this topic. A strength of our approach is that it was multidisciplinary, involving researchers and databases from the fields of health and technology. Incorporating ACM as an extra source of data widened the scope of this review significantly. It also revealed that multiarmed randomized trials are far less common in this database, and eHealth evaluations tend to be more qualitative and iterative in smaller samples. In addition, we followed the PRISMA guidelines for systematic studies.

When interpreting the results of the review, the following limitations need to be considered. Because we initially planned to report on study health outcomes in relation to tailoring, we only included interventions that were used in a randomized trial. This sometimes resulted in the exclusion of interesting studies, such as research protocols, (speculative) design studies, and single-armed efficacy tests or pilot studies. However, limiting the included studies to multiarmed trials also entailed a certain level of product maturity, compared with more speculative or single-arm pilot studies. This is probably one reason why the quality of the included studies was moderate to high.

In addition, we initially aimed to study the comparative effectiveness of different tailoring strategies to optimize intervention outcomes; however, the degree of variation among the selected studies made this infeasible. A comparative effectiveness study is recommended for future research.

Another limitation is that we were dependent on the quality and focus of reporting. If a design rationale is not described, this does not necessarily mean that it was not present or part of the development process. The constraints of this review limited us from studying other publications linked to the intervention, for instance, study protocols or design studies where more information on the intervention’s design rationale could have been present. In any case, our study suggests that design rationales for digital interventions are often not reported or even summarized in efficacy studies, implying that there are limited possibilities to be informed or inspired by approaches and choices of other researchers published in the scientific literature.

The quality of included studies in this review was moderate to high. For future studies, quality can be improved by improved samples selection and by a more critical discussion of strengths and limitations. Of course, QuADS focuses on the study design in general and whether useful conclusions can be drawn from the outcomes, not so much on whether the intervention itself was well designed or if the choices made in the design process are justifiable. As discussed, there may be opportunities to explicate standards on reporting that include (summaries of) design rationales for eHealth interventions.

### Conclusion

In conclusion, we can see a broad variety of approaches to the use of health literacy concepts and tailoring strategies in tailored eHealth interventions. We identified rationales for choices of components in all studies, and in a small minority, we could discern overarching design rationales. Although we have been able to discern health literacy concepts, tailoring strategies, and design rationales in the interventions, only a few studies provide sufficient insight into the inner workings and overarching rationale behind the intervention. A significant number of studies refers to other sources for details on the design and design process of the intervention, and while this strategy of reporting on the origins of an intervention certainly is preferable over no reporting at all, it does provide an extra barrier in linking intervention design choices to observed outcomes. Indeed, existing frameworks already provide opportunities to consistently report on the design of tailored health interventions and could be used to report on the use of cognitive and social health literacy concepts as well.

In the field of human-computer interaction, extensive reporting on the design process, as well as describing an overarching rationale for a product, is far more common. Literature has already called for a stricter reporting standard on tailored interventions [10], and since these recommendations are not specifically aimed at digital interventions, there is room to iterate on these recommendations with digital health in mind. Indeed, there are clear opportunities to explore novel reporting frameworks that incorporate the key design rationales of eHealth interventions, in addition to their efficacy. If such frameworks are used regularly, it will be feasible to study comparative effectiveness of eHealth interventions with design rationales informed by specific tailoring strategies. Future studies should therefore prioritize comprehensive documentation on the design of the intervention to facilitate replicability and understanding of intervention efficacy.

## Supplementary material

10.2196/76172 Multimedia Appendix 1 Table S1: Search strategy per database.

10.2196/76172 Multimedia Appendix 2 Table S1: general characteristics.

10.2196/76172 Multimedia Appendix 3 Table S1: intervention descriptions and components.

10.2196/76172 Multimedia Appendix 4 Table S1: QuADS scoring results.

10.2196/76172 Checklist 1 Preferred Reporting Items for Systematic Reviews and Meta-Analyses 2020 checklist as requested.

## Abbreviations

ACM: Association for Computing Machinery
HLS-EU: European Health Literacy Survey
IBIS: Issue Based Information Systems
PRISMA: Preferred Reporting Items for Systematic Reviews and Meta-Analyses
PROSPERO: International Prospective Register of Systematic Reviews
QuADS: Quality Assessment for Diverse Studies
