# Supplementary material for: Tailoring for Health Literacy in the Design and Development of eHealth Interventions: Systematic Review
Source: JMIR Hum Factors. 2025 Sep 2;12:e76172. doi: 10.2196/76172 (PMC12404580; doi:10.2196/76172)
Supplement: Multimedia Appendix 1 [file humanfactors-v12-e76172-s001.docx]

| **Table S1: Search Queries per Database** | | | | | |
| --- | --- | --- | --- | --- | --- |
| **Concept** | **Pubmed Query** | **Psychinfo** | **ACM** | **Web Of Science** | **Total** |
| **1. eHealth** | (ehealth[tiab] OR e-health[tiab] OR website*[tiab] OR web[tiab] OR webbased[tiab] OR online[tiab] OR internet[tiab] OR “mobile health” [tiab] OR “mhealth” [tiab] OR “m-health” [tiab] OR “internet intervention*”[tiab] OR “online intervention*”[tiab] OR “online program*”[tiab] OR “internet program*”[tiab] OR “online treatment*”[tiab] OR “internet treatment*”[tiab] OR (Health[tiab] AND (App [tiab] OR apps[tiab] OR Application[tiab]))) | (ehealth.mp OR e-health.mp OR website*.mp OR web.mp OR webbased.mp OR online.mp OR internet.mp OR mobile health.mp OR mhealth.mp OR m-health.mp OR internet intervention*.mp OR online intervention*.mp OR online program*.mp OR internet program*.mp OR online treatment*.mp OR internet treatment*.mp OR (Health.mp AND (App.mp OR apps.mp OR Application.mp))) | Abstract:((ehealth OR e\-health OR website* OR web OR webbased OR online OR internet OR mobile health OR mhealth OR m\-health OR "internet intervention" OR "internet interventions" OR "online intervention" OR "online interventions" OR "online Program" OR "internet program" OR "online treatment" OR "online treatments" OR "internet treatment" OR "internet treatments" OR (Health AND (App OR apps OR Application)))) | AB=(ehealth OR e\-health OR website* OR web OR webbased OR online OR internet OR mobile health OR mhealth OR m\-health OR "internet intervention" OR "internet interventions" OR "online intervention" OR "online interventions" OR "online Program" OR "internet program" OR "online treatment" OR "online treatments" OR "internet treatment" OR "internet treatments" OR (Health AND (App OR apps OR Application))) |  |
| **2. Health literacy social & cognitive concepts** | (“Patient activation” OR self-efficacy [tiab] OR motivat*[tiab] OR "Health Literacy"[Mesh] OR "Health Literacy"[tiab] OR "health knowledge*"[tiab] OR "medical data interpretation*"[tiab] OR "health competence*"[tiab]) OR (("Health"[Mesh] OR health[tiab] OR patient*) AND (literacy[tiab] OR literate[tiab] OR "reading skill*"[tiab] OR "reading abilit*"[tiab] OR "reading level*"[tiab] OR "writing level*"[tiab] OR "writing abilit*"[tiab] OR "writing skill*"[tiab] OR numeracy[tiab] OR analphabetism[tiab])) | ((Patient activation.mp OR self-efficacy.mp OR motivat*.mp OR Health Literacy.mp OR health knowledge*.mp OR medical data interpretation*.mp OR health competence*.mp) OR ((health.mp OR patient*.mp) AND (literacy.mp OR literate.mp OR reading skill*.mp OR reading abilit*.mp OR reading level*.mp OR writing level*.mp OR writing abilit*.mp OR writing skill*.mp OR numeracy.mp OR analphabetism.mp))) | Abstract:((“Patient activation” OR self\-efficacy OR motivat* OR "Health Literacy" OR "health knowledge" OR "medical data interpretation" OR "medical data interpretations" OR "health competence" OR "health competences") OR ((health OR patient*) AND (literacy OR literate OR "reading skill" OR "reading skills" OR "reading ability" OR "reading abilities" OR "reading level" OR "reading levels" OR "writing level" OR "writing levels" OR "writing ability" OR "writing abilities" OR "writing skill" OR "writing skills" OR numeracy OR analphabetism))) | AB=(("Patient activation" OR "self-efficacy" OR motivat* OR "Health Literacy" OR "health knowledge*" OR "medical data interpretation" OR "medical data interpretations" OR "health competence" OR "health competences") OR ((health OR patient*) AND (literacy OR literate OR "reading skill" OR "reading skills" OR "reading ability" OR "reading abilities" OR "reading level" OR "reading levels" OR "writing level" OR "writing levels" OR "writing ability" OR "writing abilities" OR "writing skill" OR "writing skills" OR numeracy OR analphabetism))) |  |
| **3. Tailoring** | (personalis* OR personaliz* OR individuali* OR tailor* OR custom*) | (personalis* OR personaliz* OR individuali* OR tailor* OR custom*).mp | AllField:((personalis* OR personaliz* OR individuali* OR tailor* OR custom*)) | ALL=(personalis* OR personaliz* OR individuali* OR tailor* OR custom*) |  |
| **Results 1 AND 2 AND 3** | 3308 | 1916 | 4.403 | 3188 | 12815 |
