# Supplementary material for: Tailoring for Health Literacy in the Design and Development of eHealth Interventions: Systematic Review
Source: JMIR Hum Factors. 2025 Sep 2;12:e76172. doi: 10.2196/76172 (PMC12404580; doi:10.2196/76172)
Supplement: Multimedia Appendix 2 [file humanfactors-v12-e76172-s002.docx]

| **Table S1: General Characteristics** | | | | | |
| --- | --- | --- | --- | --- | --- |
| **Author** | **Year** | **Country** | **Title** | **Illness / Behavioural Group** |  |
| Barnabei et al [23] | 2008 | USA | The Effects of a Web-Based Tool on Patient-Provider Communication and Satisfaction with Hormone Therapy: A Randomized Evaluation | Hormone therapy (Menopausal Symptoms) |  |
| Bomfim et al [24] | 2020 | Canada | Food Literacy while Shopping: Motivating Informed Food Purchasing Behaviour with a Situated Gameful App | Food Intake |  |
| Bommele et al [25] | 2017 | Netherlands | Targeting hardcore smokers: The effects of an online tailored intervention, based on motivational interviewing techniques | Smoking |  |
| Boyd et al [29] | 2020 | USA | Elderly Medication Adherence Intervention Using the My Interventional Drug-Eluting Stent Educational App: Multisite Randomized Feasibility Trial | Cardiovascular Disease (Myocardial Infarction) |  |
| Bromberg et al [26] | 2012 | USA | A Randomized Trial of a Web-based Intervention to Improve Migraine Self-Management and Coping | Migraine |  |
| Burgermaster et al [20] | 2017 | USA | The Role of Explanations in Casual Observational Learning about Nutrition | Food Intake |  |
| Carter-Harris et al [27] | 2020 | USA | Computer-Tailored Decision Support Tool for Lung Cancer Screening: Community-Based Pilot Randomized Controlled Trial | Cancer (Lung) |  |
| Chiauzzi et al [28] | 2010 | USA | PainACTION-Back Pain: A Self-Management Website for People with Chronic Back | Back Pain |  |
| Côté et al [21] | 2018 | Canada | Web-Based Tailored Intervention to Support Optimal Medication Adherence Among Kidney Transplant Recipients: Pilot Parallel-Group Randomized Controlled Trial | Medication adherence (Kidney Transplant) |  |
| Dingle and Carter [49] | 2017 | Australia | Smoke into Sound: A pilot randomised controlled trial of a music cravings management program for chronic smokers attempting to quit | Smoking |  |
| Drieling et al [30] | 2011 | USA | An Internet-Based Osteoporotic Fracture Risk Program: Effect on Knowledge, Attitudes, and Behaviors | Preventative Care (fractures) |  |
| Engelen et al [31] | 2020 | Netherlands | Evaluation of a Web-Based Self-Management Program for Patients With Cardiovascular Disease: Explorative Randomized Controlled Trial | Cardiovascular Disease |  |
| Flight et al [19] | 2012 | Australia | Decision Support and the Effectiveness of Web-based Delivery and Information Tailoring for Bowel Cancer Screening: An Exploratory Study | Cancer (Bowel) |  |
| Ford-Gilboe et al [32] | 2020 | Canada | Longitudinal impacts of an online safety and health intervention for women experiencing intimate partner violence: randomized controlled trial | Partner Violence |  |
| Fowler et al [33] | 2017 | USA | Using an Internet-Based Breast Cancer Risk Assessment Tool to Improve Social-Cognitive Precursors of Physical Activity | Cancer (Breast) |  |
| Gimbel et al [22] | 2020 | USA | Enhancing Patient Activation and Self-Management Activities in Patients With Type 2 Diabetes Using the US Department of Defense Mobile Health Care Environment: Feasibility Study | Diabetes  (Type 2) |  |
| Höchsmann et al [34] | 2019 | Switzerland | Effectiveness of a Behavior Change Technique-Based Smartphone Game to Improve Intrinsic Motivation and Physical Activity Adherence in Patients With Type 2 Diabetes: Randomized Controlled Trial | Diabetes  (Type 2) |  |
| Hopkin et al [35] | 2019 | UK/CA/USA | Combining Multiple Treatment Comparisons with Personalized Patient Preferences: A Randomized Trial of an Interactive Platform for Statin Treatment Selection | Preventative Care (Cholesterol) |  |
| Irvine et al [36] | 2015 | USA | Mobile-Web App to Self-Manage Low Back Pain: Randomized Controlled Trial | Back Pain |  |
| Kukafka et al [37] | 2002 | USA | Web-based Tailoring and its Effect on Self-Efficacy: Results from The MI-HEART Randomized Controlled Trial | Cardiovascular Disease (Myocardial Infarction) |  |
| Maddison et al [38] | 2015 | New Zealand | A mobile phone intervention increases physical activity in people with cardiovascular disease: Results from the HEART randomized controlled trial | Cardiovascular Disease (Ischemic Heart Disease) |  |
| Mevissen et al [39] | 2011 | Netherlands | Justify your love: Testing an online STI-risk communication intervention designed to promote condom use and STI-testing | Sexually Transmitted Infection |  |
| Middelweerd et al [40] | 2020 | Netherlands | The Use and Effects of an App-Based Physical Activity Intervention "Active2Gether" in Young Adults: Quasi-Experimental Trial | Physical Activity |  |
| Milan and White [41] | 2010 | USA | Impact of a Stage-Tailored, Web-Based Intervention on Folic Acid–Containing Multivitamin Use by College Women | Preventative Care (Vitamins intake) |  |
| Politi et al [42] | 2020 | USA | A Randomized Controlled Trial Evaluating the BREASTChoice Tool for Personalized Decision Support About Breast Reconstruction After Mastectomy | Breast Reconstruction |  |
| Sittig et al [43] | 2020 | USA | Incorporating Behavioral Trigger Messages Into a Mobile Health App for Chronic Disease Management: Randomized Clinical Feasibility Trial in Diabetes | Diabetes  (Type 2) |  |
| Valle et al [44] | 2018 | USA | Optimizing Tailored Communications for Health Risk Assessment: A Randomized Factorial Experiment of the Effects of Expectancy Priming, Autonomy Support, and Exemplification | Smoking, PA, Food Intake, Weight |  |
| Vernon et al [45] | 2011 | USA | A Randomized Controlled Trial of a Tailored Interactive Computer-Delivered Intervention to Promote Colorectal Cancer Screening: Sometimes More is Just the Same | Cancer (Bowel) |  |
| Weymann et al [46] | 2015 | Germany | Effectiveness of a Web-Based Tailored Interactive Health Communication Application for Patients With Type 2 Diabetes or Chronic Low Back Pain: Randomized Controlled Trial | Diabetes  (Type 2),  Back Pain |  |
| Wilson et al [47] | 2015 | Australia | A randomised controlled trial of personalised decision support delivered via the internet for bowel cancer screening with a faecal occult blood test: the effects of tailoring of messages according to social cognitive variables on participation | Cancer (Bowel) |  |
| Wong et al [48] | 2021 | Hong Kong | An Interactive Web-Based Sexual Health Literacy Program for Safe Sex Practice for Female Chinese University Students: Multicenter Randomized Controlled Trial | Preventative care (Sexually Transmitted Infection / Unwanted Pregnancy) |  |

# **References**

23. Barnabei VM, O'Connor JJ, Nimphius NM, Vierkant RA, Eaker ED, Ahmad. The effects of a web-based tool on patient-provider communication and satisfaction with hormone therapy: A randomized evaluation. Journal of Women's Health. 2008;17(1):147-58. doi: 10.1089/jwh.2007.0369.

24. Bomfim MCC, Kirkpatrick SI, Nacke LE, Wallace JR. Food Literacy While Shopping: Motivating Informed Food Purchasing Behaviour with a Situated Gameful App. 2020:1–13.

25. Bommele J, Schoenmakers TM, Kleinjan M, Peters G-JY, Dijkstra A, van de Mheen D, et al. Targeting hardcore smokers: The effects of an online tailored intervention, based on motivational interviewing techniques. British Journal of Health Psychology. 2017;22(3):644-60. doi: 10.1111/bjhp.12256.

29. Boyd AD, Ndukwe CI, Dileep A, Everin OF, Yao Y, Welland B, et al. Elderly Medication Adherence Intervention Using the My Interventional Drug-Eluting Stent Educational App: Multisite Randomized Feasibility Trial. JMIR Mhealth Uhealth. 2020;8(6):e15900. doi: 10.2196/15900. PubMed PMID: 32579120.

26. Bromberg J, Wood ME, Black RA, Surette DA, Zacharoff KL, Chiauzzi EJ. A randomized trial of a web-based intervention to improve migraine self-management and coping. Headache. 2012;52(2):244-61. doi: 10.1111/j.1526-4610.2011.02031.x. PubMed PMID: 22413151.

20. Burgermaster M, Gajos KZ, Davidson P, Mamykina L. The Role of Explanations in Casual Observational Learning about Nutrition. 2017:4097–145.

27. Carter-Harris L, Comer RS, Slaven Ii JE, Monahan PO, Vode E, Hanna NH, et al. Computer-Tailored Decision Support Tool for Lung Cancer Screening: Community-Based Pilot Randomized Controlled Trial. J Med Internet Res. 2020;22(11):e17050. doi: 10.2196/17050. PubMed PMID: 33141096.

28. Chiauzzi E, Pujol LA, Wood M, Bond K, Black R, Yiu E, et al. painACTION-Back Pain: A Self-Management Website for People with Chronic Back Pain. Pain Medicine. 2010;11(7):1044-58. doi: 10.1111/j.1526-4637.2010.00879.x. PubMed PMID: WOS:000279125000009.

21. Côté J, Fortin MC, Auger P, Rouleau G, Dubois S, Boudreau N, et al. Web-Based Tailored Intervention to Support Optimal Medication Adherence Among Kidney Transplant Recipients: Pilot Parallel-Group Randomized Controlled Trial. JMIR Form Res. 2018;2(2):e14. doi: 10.2196/formative.9707. PubMed PMID: 30684400.

49. Dingle GA, Carter NA. Smoke into Sound: A pilot randomised controlled trial of a music cravings management program for chronic smokers attempting to quit. Musicae Scientiae. 2017;21(2):151-77. doi: 10.1177/1029864916682822. PubMed PMID: WOS:000402004200003.

30. Drieling RL, Ma J, Thiyagarajan S, Stafford RS, Bachmann. An Internet-based osteoporotic fracture risk program: Effect on knowledge, attitudes, and behaviors. Journal of Women's Health. 2011;20(12):1895-907. doi: http://dx.doi.org/10.1089/jwh.2010.2515.

31. Engelen MM, van Dulmen S, Puijk-Hekman S, Vermeulen H, Nijhuis-van der Sanden MW, Bredie SJ, et al. Evaluation of a Web-Based Self-Management Program for Patients With Cardiovascular Disease: Explorative Randomized Controlled Trial. J Med Internet Res. 2020;22(7):e17422. doi: 10.2196/17422. PubMed PMID: 32706708.

19. Flight IH, Wilson CJ, Zajac IT, Hart E, McGillivray JA. Decision Support and the Effectiveness of Web-based Delivery and Information Tailoring for Bowel Cancer Screening: An Exploratory Study. JMIR Res Protoc. 2012;1(2):e12. doi: 10.2196/resprot.2135. PubMed PMID: 23611950.

32. Ford-Gilboe M, Varcoe C, Scott-Storey K, Perrin N, Wuest J, Wathen CN, et al. Longitudinal impacts of an online safety and health intervention for women experiencing intimate partner violence: randomized controlled trial. BMC Public Health. 2020;20(1):260. doi: 10.1186/s12889-020-8152-8. PubMed PMID: 32098633.

33. Fowler SL, Klein WMP, Ball L, McGuire J, Colditz GA, Waters EA. Using an Internet-Based Breast Cancer Risk Assessment Tool to Improve Social-Cognitive Precursors of Physical Activity. Med Decis Making. 2017;37(6):657-69. doi: 10.1177/0272989x17699835. PubMed PMID: 28363033.

22. Gimbel RW, Rennert LM, Crawford P, Little JR, Truong K, Williams JE, et al. Enhancing Patient Activation and Self-Management Activities in Patients With Type 2 Diabetes Using the US Department of Defense Mobile Health Care Environment: Feasibility Study. J Med Internet Res. 2020;22(5):e17968. doi: 10.2196/17968. PubMed PMID: 32329438.

34. Höchsmann C, Infanger D, Klenk C, Königstein K, Walz SP, Schmidt-Trucksäss A. Effectiveness of a Behavior Change Technique-Based Smartphone Game to Improve Intrinsic Motivation and Physical Activity Adherence in Patients With Type 2 Diabetes: Randomized Controlled Trial. JMIR Serious Games. 2019;7(1):e11444. doi: 10.2196/11444. PubMed PMID: 30758293.

35. Hopkin G, Au A, Collier VJ, Yudkin JS, Basu S, Naci H. Combining Multiple Treatment Comparisons with Personalized Patient Preferences: A Randomized Trial of an Interactive Platform for Statin Treatment Selection. Med Decis Making. 2019;39(3):264-77. doi: 10.1177/0272989x19835239. PubMed PMID: 30873906.

36. Irvine AB, Russell H, Manocchia M, Mino DE, Cox Glassen T, Morgan R, et al. Mobile-Web app to self-manage low back pain: randomized controlled trial. J Med Internet Res. 2015;17(1):e1. doi: 10.2196/jmir.3130. PubMed PMID: 25565416.

37. Kukafka R, Lussier YA, Eng P, Patel VL, Cimino JJ. Web-based tailoring and its effect on self-efficacy: results from the MI-HEART randomized controlled trial. Proc AMIA Symp. 2002:410-4. PubMed PMID: 12463857.

38. Maddison R, Pfaeffli L, Whittaker R, Stewart R, Kerr A, Jiang Y, et al. A mobile phone intervention increases physical activity in people with cardiovascular disease: Results from the HEART randomized controlled trial. Eur J Prev Cardiol. 2015;22(6):701-9. doi: 10.1177/2047487314535076. PubMed PMID: 24817694.

39. Mevissen FE, Ruiter RA, Meertens RM, Zimbile F, Schaalma HP. Justify your love: testing an online STI-risk communication intervention designed to promote condom use and STI-testing. Psychol Health. 2011;26(2):205-21. doi: 10.1080/08870446.2011.531575. PubMed PMID: 21318930.

40. Middelweerd A, Mollee J, Klein MM, Manzoor A, Brug J, Te Velde SJ. The Use and Effects of an App-Based Physical Activity Intervention "Active2Gether" in Young Adults: Quasi-Experimental Trial. JMIR Form Res. 2020;4(1):e12538. doi: 10.2196/12538. PubMed PMID: 31961330.

41. Milan JE, White AA. Impact of a stage-tailored, web-based intervention on folic acid-containing multivitamin use by college women. Am J Health Promot. 2010;24(6):388-95. doi: 10.4278/ajhp.071231143. PubMed PMID: 20594096.

42. Politi MC, Lee CN, Philpott-Streiff SE, Foraker RE, Olsen MA, Merrill C, et al. A Randomized Controlled Trial Evaluating the BREASTChoice Tool for Personalized Decision Support About Breast Reconstruction After Mastectomy. Ann Surg. 2020;271(2):230-7. doi: 10.1097/sla.0000000000003444. PubMed PMID: 31305282.

43. Sittig S, Wang J, Iyengar S, Myneni S, Franklin A. Incorporating Behavioral Trigger Messages Into a Mobile Health App for Chronic Disease Management: Randomized Clinical Feasibility Trial in Diabetes. JMIR Mhealth Uhealth. 2020;8(3):e15927. doi: 10.2196/15927. PubMed PMID: 32175908.

44. Valle CG, Queen TL, Martin BA, Ribisl KM, Mayer DK, Tate DF. Optimizing Tailored Communications for Health Risk Assessment: A Randomized Factorial Experiment of the Effects of Expectancy Priming, Autonomy Support, and Exemplification. J Med Internet Res. 2018;20(3):e63. doi: 10.2196/jmir.7613. PubMed PMID: 29496652.

45. Vernon SW, Bartholomew LK, McQueen A, Bettencourt JL, Greisinger A, Coan SP, et al. A randomized controlled trial of a tailored interactive computer-delivered intervention to promote colorectal cancer screening: sometimes more is just the same. Ann Behav Med. 2011;41(3):284-99. doi: 10.1007/s12160-010-9258-5. PubMed PMID: 21271365.

45. Weymann N, Dirmaier J, von Wolff A, Kriston L, Harter M, Airaksinen. Effectiveness of a Web-based tailored interactive health communication application for patients with type 2 diabetes or chronic low back pain: Randomized controlled trial. Journal of Medical Internet Research. 2015;17(3):No-Specified. doi: 10.2196/jmir.3904.

47. Wilson CJ, Flight IH, Turnbull D, Gregory T, Cole SR, Young GP, et al. A randomised controlled trial of personalised decision support delivered via the internet for bowel cancer screening with a faecal occult blood test: the effects of tailoring of messages according to social cognitive variables on participation. BMC Med Inform Decis Mak. 2015;15:25. doi: 10.1186/s12911-015-0147-5. PubMed PMID: 25886492.

48. Wong JY, Zhang W, Wu Y, Choi EPH, Lo HHM, Wong W, et al. An Interactive Web-Based Sexual Health Literacy Program for Safe Sex Practice for Female Chinese University Students: Multicenter Randomized Controlled Trial. J Med Internet Res. 2021;23(3):e22564. doi: 10.2196/22564. PubMed PMID: 33709941.
