# Supplementary material for: Tailoring for Health Literacy in the Design and Development of eHealth Interventions: Systematic Review
Source: JMIR Hum Factors. 2025 Sep 2;12:e76172. doi: 10.2196/76172 (PMC12404580; doi:10.2196/76172)
Supplement: Multimedia Appendix 3 [file humanfactors-v12-e76172-s003.docx]

| **Table S1: Intervention Descriptions & Components** | | | |
| --- | --- | --- | --- |
| **Author** | **Intervention Description and Intent / Objective** | **Media/Technology** | **Component(s)** |
| Barnabei et al [23] | "The intent of [the Internet-based questionnaire] is [to provide information and customized questions] to facilitate more informative, shared, and efficient patient-provider communication and not to provide medical advice." | Website; Desktop; Print | eLearning; Questionnaire; Automated feedback |
| Bomfim et al [24] | A Situated Gameful App "to motivate young adults to develop food literacy, increase awareness, and improve choices at the grocery store." | App; Mobile | Diary / Logging; Messaging; eLearning; Game / Game mechanics; Automated feedback |
| Bommele et al [25] | The use of a virtual online character offering three interventions “to increase hardcore smokers’ willingness to read tobacco control messages and to increase hardcore smokers’ intention to quit and their receptivity to information about smoking cessation" | App; Desktop | Messaging; eLearning; Questionnaire; Virtual agent; Automated feedback |
| Boyd et al [29] | A computing tablet-based education program to educate patients about the importance of dual antiplatelet therapy. | App; Tablet | Diary / Logging; eLearning; Questionnaire |
| Bromberg et al [26] | A website to " increase patient self-efficacy to perform headache self-management activities and symptom management strategies; and reduce migraine-related psychological distress." | Website; E-mail | Diary / Logging; Messaging; eLearning; Questionnaire; Onboarding |
| Burgermaster et al [20] | An online Nutrition Knowledge Test to "test knowledge on nutrition" | Website | eLearning; Questionnaire; Automated feedback |
| Carter-Harris et al [27] | a computer-tailored decision support tool to “support shared decision making in lung cancer screening decisions" | Website; Desktop | eLearning; Questionnaire; Automated feedback |
| Chiauzzi et al [28] | A self-management website to "improve emotional management, coping, self-efficacy to manage pain, pain levels, and physical functioning" | Website | eLearning; Questionnaire; Automated feedback |
| Côté et al [21] | Three interactive Web-based sessions hosted by a virtual nurse, "strengthening various capacities such as self-motivation and self-monitoring, problem solving and emotional control, and social interaction." | Website | eLearning; Virtual agent; Automated feedback |
| Dingle and Carter[49] | Website that teaches "participants to identify which emotions were triggers for their smoking" | Website | Diary / Logging; eLearning |
| Drieling et al [30] | Internet-based health education program to promote self-management of fracture risk | Website; E-mail | Messaging; eLearning; Questionnaire; Automated feedback |
| Engelen et al [31] | A web-based self-management program "to increase self-management behavior by tailoring to the perceived problems and (support) needs of patients after CVD" | Website | Diary / Logging; Messaging; eLearning; Questionnaire; Automated feedback |
| Flight et al [19] | A website "to provide information tailored to an individual’s decision stage for CRC screening and attitude toward screening utilizing the Preventive Health Model (PHM)" | Website | Messaging; eLearning; Questionnaire; Automated feedback |
| Ford-Gilboe et al [32] | A website "to increase (women's) awareness of safety risks and reflect on their plans for their relationships and priorities" | Website | eLearning; Questionnaire; Automated feedback |
| Fowler et al [33] | A website to "elicit higher levels of physical activity–related behavioral intentions, self-efficacy, and response efficacy." | Website | eLearning; Questionnaire; Automated feedback |
| Gimbel et al [22] | A health portal accessed by mobile phones and tablets "to enhance patient activation and self-management of T2D using the US Department of Defense’s Mobile Health Care Environment (MHCE) in a patient-centered medical home setting" | App; Mobile; Wearable; SMS | Diary / Logging; Messaging; Automated feedback; Dashboard |
| Höchsmann et al [34] | A behavior changes technique-based smartphone game "to motivate inactive individuals with type 2 diabetes for regular use and thereby increase their intrinsic PA motivation" | App; Mobile | Diary / Logging; eLearning; Questionnaire; Game / Game mechanics; Automated feedback; Onboarding; Dashboard |
| Hopkin et al [35] | A SEUT-based webtool that "synthesizes key findings from published network meta analyses on the benefits and tolerability and harms of the five most commonly used cholesterol-lowering treatments, statins" | Website | Questionnaire; Automated feedback; Onboarding |
| Irvine et al [36] | "A mobile-Web intervention called “FitBack” to help users implement self-tailored strategies to manage and prevent NLBP occurrences" | Website; Mobile; Tablet; Desktop; E-mail | Diary / Logging; Messaging; eLearning; Questionnaire; Virtual agent; Dashboard |
| Kukafka et al [37] | "A clinical information system [to] favorably influence the appropriateness and rapidity of decision-making in patients suffering from symptoms of acute myocardial infarction." | Website | Messaging; eLearning; Questionnaire; Automated feedback |
| Maddison et al [38] | "A mobile phone intervention to support. The intervention focused on increasing leisure time physical activity – walking in particular – as well as encouragement to accrue incidental activity through daily tasks such as household chores and active transport for people with ischaemic heart disease (IHD)." | Website; Mobile; SMS | Diary / Logging; Messaging; eLearning; Automated feedback; Dashboard |
| Mevissen et al [39] | "A tailored, web-based intervention [virtual STI public clinic] communicating the risks of sexually transmitted infections (STI) for heterosexual young adults, to stimulate maintenance of condom use and to promote STI-testing " | Website | eLearning; Questionnaire; Virtual agent; Automated feedback |
| Middelweerd et al [40] | An "app-based intervention (Active2Gether) to increase the levels of PA in young adults" | App; Mobile; Wearable; SMS | Diary / Logging; Messaging; Game / Game mechanics; Automated feedback; Dashboard |
| Milan and White [41] | A "web-based, stage-tailored folic acid intervention [...]to promote folic acid-containing multivitamin use among college women." | Website; E-mail | eLearning; Questionnaire |
| Politi et al [42] | a web-based breast reconstruction decision aid to improve breast reconstruction decision quality by improving patients’ knowledge and providing them with personalized risk estimates. | Website; Print | eLearning; Questionnaire; Automated feedback |
| Sittig et al [43] | "...an engaged mHealth app focused on behavioral change to improve self-efficacy, knowledge, and self-management for individuals with chronic disease" | App; Mobile; Messaging or video service; SMS | Diary / Logging; Messaging; eLearning; Questionnaire; Game / Game mechanics; Automated feedback; Onboarding |
| Valle et al [44] | to promote health behavior, change through feedback messages within an existing Health Assessment and resource tool, tailored for expectancy priming, autonomy support, and use of an exemplar. | Website | Messaging; eLearning; Questionnaire; Virtual agent; Automated feedback |
| Vernon et al [45] | A tailored, interactive intervention to increase CRC screening | Website; Print | Messaging; eLearning; Questionnaire; Automated feedback |
| Weymann et al [46] | "A tailored IHCA presenting information on T2D and CLBP, self-management education, and decision support, focusing on more proximal outcomes such as health-related empowerment and knowledge" | Website | eLearning; Questionnaire; Automated feedback; Onboarding |
| Wilson et al [47] | An internet-based, tailored, Personalised Decision Support (PDS) tool for bowel cancer screening, tailoring health information to the needs of the individual may facilitate desired changes in intention and behaviour, including cancer screening. | Website | Messaging; eLearning |
| Wong et al [48] | "An interactive web-based intervention that aims to disseminate knowledge about STIs and condom use, communication and negotiation about condom use, and sexual coercion in daily life to enhance safe sex practices" | Website; SMS; E-mail | eLearning; Questionnaire; Automated feedback |

23. Barnabei VM, O'Connor JJ, Nimphius NM, Vierkant RA, Eaker ED, Ahmad. The effects of a web-based tool on patient-provider communication and satisfaction with hormone therapy: A randomized evaluation. Journal of Women's Health. 2008;17(1):147-58. doi: 10.1089/jwh.2007.0369.

24. Bomfim MCC, Kirkpatrick SI, Nacke LE, Wallace JR. Food Literacy While Shopping: Motivating Informed Food Purchasing Behaviour with a Situated Gameful App. 2020:1–13.

25. Bommele J, Schoenmakers TM, Kleinjan M, Peters G-JY, Dijkstra A, van de Mheen D, et al. Targeting hardcore smokers: The effects of an online tailored intervention, based on motivational interviewing techniques. British Journal of Health Psychology. 2017;22(3):644-60. doi: 10.1111/bjhp.12256.

29. Boyd AD, Ndukwe CI, Dileep A, Everin OF, Yao Y, Welland B, et al. Elderly Medication Adherence Intervention Using the My Interventional Drug-Eluting Stent Educational App: Multisite Randomized Feasibility Trial. JMIR Mhealth Uhealth. 2020;8(6):e15900. doi: 10.2196/15900. PubMed PMID: 32579120.

26. Bromberg J, Wood ME, Black RA, Surette DA, Zacharoff KL, Chiauzzi EJ. A randomized trial of a web-based intervention to improve migraine self-management and coping. Headache. 2012;52(2):244-61. doi: 10.1111/j.1526-4610.2011.02031.x. PubMed PMID: 22413151.

20. Burgermaster M, Gajos KZ, Davidson P, Mamykina L. The Role of Explanations in Casual Observational Learning about Nutrition. 2017:4097–145.

27. Carter-Harris L, Comer RS, Slaven Ii JE, Monahan PO, Vode E, Hanna NH, et al. Computer-Tailored Decision Support Tool for Lung Cancer Screening: Community-Based Pilot Randomized Controlled Trial. J Med Internet Res. 2020;22(11):e17050. doi: 10.2196/17050. PubMed PMID: 33141096.

28. Chiauzzi E, Pujol LA, Wood M, Bond K, Black R, Yiu E, et al. painACTION-Back Pain: A Self-Management Website for People with Chronic Back Pain. Pain Medicine. 2010;11(7):1044-58. doi: 10.1111/j.1526-4637.2010.00879.x. PubMed PMID: WOS:000279125000009.

21. Côté J, Fortin MC, Auger P, Rouleau G, Dubois S, Boudreau N, et al. Web-Based Tailored Intervention to Support Optimal Medication Adherence Among Kidney Transplant Recipients: Pilot Parallel-Group Randomized Controlled Trial. JMIR Form Res. 2018;2(2):e14. doi: 10.2196/formative.9707. PubMed PMID: 30684400.

49. Dingle GA, Carter NA. Smoke into Sound: A pilot randomised controlled trial of a music cravings management program for chronic smokers attempting to quit. Musicae Scientiae. 2017;21(2):151-77. doi: 10.1177/1029864916682822. PubMed PMID: WOS:000402004200003.

30. Drieling RL, Ma J, Thiyagarajan S, Stafford RS, Bachmann. An Internet-based osteoporotic fracture risk program: Effect on knowledge, attitudes, and behaviors. Journal of Women's Health. 2011;20(12):1895-907. doi: http://dx.doi.org/10.1089/jwh.2010.2515.

31. Engelen MM, van Dulmen S, Puijk-Hekman S, Vermeulen H, Nijhuis-van der Sanden MW, Bredie SJ, et al. Evaluation of a Web-Based Self-Management Program for Patients With Cardiovascular Disease: Explorative Randomized Controlled Trial. J Med Internet Res. 2020;22(7):e17422. doi: 10.2196/17422. PubMed PMID: 32706708.

19. Flight IH, Wilson CJ, Zajac IT, Hart E, McGillivray JA. Decision Support and the Effectiveness of Web-based Delivery and Information Tailoring for Bowel Cancer Screening: An Exploratory Study. JMIR Res Protoc. 2012;1(2):e12. doi: 10.2196/resprot.2135. PubMed PMID: 23611950.

32. Ford-Gilboe M, Varcoe C, Scott-Storey K, Perrin N, Wuest J, Wathen CN, et al. Longitudinal impacts of an online safety and health intervention for women experiencing intimate partner violence: randomized controlled trial. BMC Public Health. 2020;20(1):260. doi: 10.1186/s12889-020-8152-8. PubMed PMID: 32098633.

33. Fowler SL, Klein WMP, Ball L, McGuire J, Colditz GA, Waters EA. Using an Internet-Based Breast Cancer Risk Assessment Tool to Improve Social-Cognitive Precursors of Physical Activity. Med Decis Making. 2017;37(6):657-69. doi: 10.1177/0272989x17699835. PubMed PMID: 28363033.

22. Gimbel RW, Rennert LM, Crawford P, Little JR, Truong K, Williams JE, et al. Enhancing Patient Activation and Self-Management Activities in Patients With Type 2 Diabetes Using the US Department of Defense Mobile Health Care Environment: Feasibility Study. J Med Internet Res. 2020;22(5):e17968. doi: 10.2196/17968. PubMed PMID: 32329438.

34. Höchsmann C, Infanger D, Klenk C, Königstein K, Walz SP, Schmidt-Trucksäss A. Effectiveness of a Behavior Change Technique-Based Smartphone Game to Improve Intrinsic Motivation and Physical Activity Adherence in Patients With Type 2 Diabetes: Randomized Controlled Trial. JMIR Serious Games. 2019;7(1):e11444. doi: 10.2196/11444. PubMed PMID: 30758293.

35. Hopkin G, Au A, Collier VJ, Yudkin JS, Basu S, Naci H. Combining Multiple Treatment Comparisons with Personalized Patient Preferences: A Randomized Trial of an Interactive Platform for Statin Treatment Selection. Med Decis Making. 2019;39(3):264-77. doi: 10.1177/0272989x19835239. PubMed PMID: 30873906.

36. Irvine AB, Russell H, Manocchia M, Mino DE, Cox Glassen T, Morgan R, et al. Mobile-Web app to self-manage low back pain: randomized controlled trial. J Med Internet Res. 2015;17(1):e1. doi: 10.2196/jmir.3130. PubMed PMID: 25565416.

37. Kukafka R, Lussier YA, Eng P, Patel VL, Cimino JJ. Web-based tailoring and its effect on self-efficacy: results from the MI-HEART randomized controlled trial. Proc AMIA Symp. 2002:410-4. PubMed PMID: 12463857.

38. Maddison R, Pfaeffli L, Whittaker R, Stewart R, Kerr A, Jiang Y, et al. A mobile phone intervention increases physical activity in people with cardiovascular disease: Results from the HEART randomized controlled trial. Eur J Prev Cardiol. 2015;22(6):701-9. doi: 10.1177/2047487314535076. PubMed PMID: 24817694.

39. Mevissen FE, Ruiter RA, Meertens RM, Zimbile F, Schaalma HP. Justify your love: testing an online STI-risk communication intervention designed to promote condom use and STI-testing. Psychol Health. 2011;26(2):205-21. doi: 10.1080/08870446.2011.531575. PubMed PMID: 21318930.

40. Middelweerd A, Mollee J, Klein MM, Manzoor A, Brug J, Te Velde SJ. The Use and Effects of an App-Based Physical Activity Intervention "Active2Gether" in Young Adults: Quasi-Experimental Trial. JMIR Form Res. 2020;4(1):e12538. doi: 10.2196/12538. PubMed PMID: 31961330.

41. Milan JE, White AA. Impact of a stage-tailored, web-based intervention on folic acid-containing multivitamin use by college women. Am J Health Promot. 2010;24(6):388-95. doi: 10.4278/ajhp.071231143. PubMed PMID: 20594096.

42. Politi MC, Lee CN, Philpott-Streiff SE, Foraker RE, Olsen MA, Merrill C, et al. A Randomized Controlled Trial Evaluating the BREASTChoice Tool for Personalized Decision Support About Breast Reconstruction After Mastectomy. Ann Surg. 2020;271(2):230-7. doi: 10.1097/sla.0000000000003444. PubMed PMID: 31305282.

43. Sittig S, Wang J, Iyengar S, Myneni S, Franklin A. Incorporating Behavioral Trigger Messages Into a Mobile Health App for Chronic Disease Management: Randomized Clinical Feasibility Trial in Diabetes. JMIR Mhealth Uhealth. 2020;8(3):e15927. doi: 10.2196/15927. PubMed PMID: 32175908.

44. Valle CG, Queen TL, Martin BA, Ribisl KM, Mayer DK, Tate DF. Optimizing Tailored Communications for Health Risk Assessment: A Randomized Factorial Experiment of the Effects of Expectancy Priming, Autonomy Support, and Exemplification. J Med Internet Res. 2018;20(3):e63. doi: 10.2196/jmir.7613. PubMed PMID: 29496652.

45. Vernon SW, Bartholomew LK, McQueen A, Bettencourt JL, Greisinger A, Coan SP, et al. A randomized controlled trial of a tailored interactive computer-delivered intervention to promote colorectal cancer screening: sometimes more is just the same. Ann Behav Med. 2011;41(3):284-99. doi: 10.1007/s12160-010-9258-5. PubMed PMID: 21271365.

46. Weymann N, Dirmaier J, von Wolff A, Kriston L, Harter M, Airaksinen. Effectiveness of a Web-based tailored interactive health communication application for patients with type 2 diabetes or chronic low back pain: Randomized controlled trial. Journal of Medical Internet Research. 2015;17(3):No-Specified. doi: 10.2196/jmir.3904.

47. Wilson CJ, Flight IH, Turnbull D, Gregory T, Cole SR, Young GP, et al. A randomised controlled trial of personalised decision support delivered via the internet for bowel cancer screening with a faecal occult blood test: the effects of tailoring of messages according to social cognitive variables on participation. BMC Med Inform Decis Mak. 2015;15:25. doi: 10.1186/s12911-015-0147-5. PubMed PMID: 25886492.

48. Wong JY, Zhang W, Wu Y, Choi EPH, Lo HHM, Wong W, et al. An Interactive Web-Based Sexual Health Literacy Program for Safe Sex Practice for Female Chinese University Students: Multicenter Randomized Controlled Trial. J Med Internet Res. 2021;23(3):e22564. doi: 10.2196/22564. PubMed PMID: 33709941.
