# Supplementary material for: Tailoring for Health Literacy in the Design and Development of eHealth Interventions: Systematic Review
Source: JMIR Hum Factors. 2025 Sep 2;12:e76172. doi: 10.2196/76172 (PMC12404580; doi:10.2196/76172)
Supplement: Multimedia Appendix 4 [file humanfactors-v12-e76172-s004.docx]

| **Table S1: QuADS Scoring Results** | | | | | | | | | | | | | | |
| --- | --- | --- | --- | --- | --- | --- | --- | --- | --- | --- | --- | --- | --- | --- |
| **ID** | **C.1^1^** | **C.2^2^** | **C.3^3^** | **C.4^4^** | **C.5^5^** | **C.6^6^** | **C.7^7^** | **C.8^8^** | **C.9^9^** | **C.10^10^** | **C.11^11^** | **C.12^12^** | **C.13^13^** | **Total** |
| **1** | 1 | 3 | 3 | 2 | 2 | 3 | 2 | 2 | 3 | 3 | 3 | 3 | 2 | 82 % |
| **2** | 3 | 3 | 2 | 3 | 2 | 3 | 3 | 2 | 1 | 2 | 3 | 2 | 2 | 79 % |
| **3** | 3 | 2 | 2 | 1 | 2 | 1 | 2 | 3 | 3 | 1 | 2 | 0 | 2 | 62% |
| **4** | 3 | 3 | 1 | 3 | 2 | 3 | 3 | 3 | 3 | 3 | 3 | 2 | 2 | 87 % |
| **5** | 3 | 2 | 1 | 3 | 0 | 3 | 1 | 3 | 1 | 1 | 2 | 2 | 1 | 59% |
| **6** | 3 | 2 | 2 | 3 | 2 | 3 | 3 | 3 | 3 | 2 | 2 | 3 | 2 | 85% |
| **7** | 1 | 3 | 1 | 3 | 2 | 3 | 3 | 2 | 3 | 2 | 3 | 3 | 2 | 79 % |
| **8** | 2 | 2 | 3 | 3 | 2 | 3 | 3 | 3 | 3 | 2 | 3 | 3 | 2 | 87% |
| **9** | 1 | 2 | 3 | 2 | 1 | 1 | 3 | 3 | 3 | 1 | 2 | 3 | 2 | 69% |
| **10** | 3 | 3 | 2 | 3 | 2 | 2 | 3 | 3 | 3 | 2 | 2 | 3 | 2 | 85% |
| **11** | 3 | 2 | 3 | 3 | 2 | 2 | 2 | 2 | 3 | 2 | 2 | 3 | 2 | 79 % |
| **12** | 1 | 1 | 2 | 2 | 1 | 1 | 2 | 2 | 3 | 2 | 3 | 3 | 2 | 64% |
| **13** | 3 | 3 | 2 | 3 | 1 | 3 | 3 | 2 | 2 | 3 | 3 | 3 | 2 | 852% |
| **14** | 3 | 3 | 3 | 3 | 1 | 3 | 3 | 2 | 3 | 2 | 3 | 3 | 2 | 87% |
| **15** | 3 | 3 | 2 | 3 | 2 | 3 | 3 | 2 | 2 | 3 | 2 | 2 | 2 | 82% |
| **16** | 3 | 3 | 3 | 3 | 2 | 3 | 3 | 2 | 2 | 2 | 2 | 3 | 2 | 85% |
| **17** | 3 | 3 | 2 | 3 | 1 | 2 | 3 | 2 | 3 | 2 | 2 | 3 | 2 | 79% |
| **18** | 1 | 2 | 1 | 2 | 1 | 2 | 2 | 2 | 1 | 1 | 2 | 3 | 2 | 56,% |
| **19** | 2 | 3 | 1 | 3 | 2 | 3 | 3 | 2 | 3 | 2 | 2 | 1 | 2 | 74% |
| **20** | 3 | 2 | 1 | 3 | 1 | 3 | 3 | 1 | 1 | 1 | 3 | 3 | 1 | 67% |
| **21** | 3 | 3 | 2 | 3 | 2 | 2 | 3 | 2 | 3 | 2 | 2 | 3 | 2 | 82% |
| **22** | 2 | 3 | 2 | 2 | 2 | 3 | 3 | 2 | 3 | 1 | 2 | 3 | 2 | 77% |
| **23** | 1 | 2 | 1 | 2 | 1 | 2 | 2 | 2 | 3 | 1 | 2 | 3 | 2 | 62% |
| **24** | 3 | 3 | 3 | 3 | 2 | 3 | 3 | 2 | 2 | 2 | 3 | 1 | 2 | 82% |
| **25** | 2 | 3 | 3 | 2 | 2 | 2 | 2 | 3 | 3 | 2 | 2 | 3 | 1 | 77% |
| **26** | 2 | 2 | 2 | 1 | 1 | 2 | 2 | 2 | 1 | 3 | 2 | 2 | 1 | 59% |
| **27** | 3 | 3 | 1 | 3 | 2 | 2 | 3 | 2 | 2 | 3 | 3 | 2 | 2 | 79% |
| **28** | 2 | 3 | 3 | 3 | 2 | 3 | 3 | 3 | 3 | 3 | 1 | 2 | 2 | 85% |
| **29** | 3 | 3 | 2 | 2 | 3 | 3 | 3 | 2 | 3 | 2 | 2 | 3 | 2 | 85% |
| **30** | 3 | 3 | 2 | 3 | 2 | 3 | 2 | 3 | 3 | 3 | 2 | 2 | 1 | 82% |
| **31** | 3 | 2 | 2 | 3 | 2 | 3 | 3 | 2 | 3 | 3 | 2 | 2 | 2 | 82% |
| **Total** | 81% | 86% | 68% | 87% | 56% | 84% | 88% | 76% | 84% | 69% | 77% | 83% | 61% |  |

**Notes**

^1^) C.1: Theoretical or conceptual underpinning to the research
^2^) C.2: Statement of research aim/s
^3^) C.3: Clear description of research setting and target population
^4^) C.4: The study design is appropriate to address the stated research aim/s
^5^) C.5: Appropriate sampling to address the research aim/s
^6^) C.6: Rationale for choice of data collection tool/s
^7^) C.7: The format and content of data collection tool is appropriate to address the stated research aim/s
^8^) C.8: Description of data collection procedure
^9^) C.9: Recruitment data provided
^10^) C.10: Justification for analytic method selected
^11^) C.11: The method of analysis was appropriate to answer the research aim/s
^12^) C.12: Evidence that the research stakeholders have been considered in research design or conduct.
^13^) C.13: Strengths and limitations critically discussed
